# Acquired Oral Hyperpigmentation: A Benign Finding or a Malignant Clue?

**DOI:** 10.7759/cureus.100640

**Published:** 2026-01-02

**Authors:** Pablo Belmont Nava, Carol E Marquez Maldonado, Lucia Achell Nava, Dolores Maribel Arellano Vivero, Guadalupe Maldonado-Colin, Claudia L Shoup Fierro

**Affiliations:** 1 Department of Dermatology, Centro Medico Nacional 20 de Noviembre, Mexico City, MEX; 2 Faculty of Health Sciences, Centro Médico Nacional 20 de Noviembre, Mexico City, MEX; 3 Department of Dermatology, Centro Medico Nacional 20 de Noviembre, Instituto de Seguridad y Servicios Sociales de los Trabajadores del Estado, Mexico City, MEX; 4 Department of Dermatology, Hospital General Dr. Manuel Gea González, Mexico City, MEX; 5 Department of Pathology, Centro Medico Nacional 20 de Noviembre, Mexico City, MEX

**Keywords:** differential diagnosis, laugier-hunziker syndrome, melanonychia, oral hyperpigmentation, oral pigmentation

## Abstract

Laugier-Hunziker syndrome (LHS) is a rare, acquired pigmentary disorder characterized by mucocutaneous hyperpigmentation, primarily affecting the oral mucosa and nails. We present a case of a 76-year-old male patient with extensive melanotic macules on the oral mucosa, lips, tongue, genitalia, and acral regions. Histopathological examination revealed basal layer hyperpigmentation and dermal melanophages. Systemic involvement was excluded through interdisciplinary evaluation. LHS, though benign, requires differentiation from syndromes with malignant potential, such as Peutz-Jeghers syndrome. This case underscores the importance of recognizing LHS to avoid unnecessary interventions and ensure appropriate patient reassurance.

## Introduction

Laugier-Hunziker syndrome (LHS) is a rare, acquired, and benign pigmentary disorder characterized by diffuse macular hyperpigmentation of the oral mucosa and associated longitudinal melanonychia that is most commonly observed in middle-aged women [1]. In 1970, Laugier and Hunziker reported five French cases of acquired macular hyperpigmentation with no underlying disease, and two patients displayed longitudinal pigmented streaks on the nails. To date, no more than 200 cases have been reported in the literature [2].

The exact etiology and pathogenesis remain unclear and no association with malignancy or systemic disease has been noted. Although uncommon, LHS represents one of the most frequent syndromic causes of oral melanotic macules, making its recognition essential to avoid unnecessary investigations and misdiagnosis of conditions with systemic implications [1-4]. We present the case of an elderly patient with extensive mucosal pigmentation in whom, after thorough evaluation, a diagnosis of LHS was established [4].

## Case presentation

**Figure 1:**
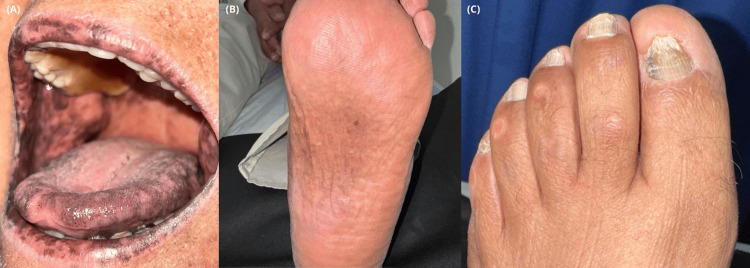
Clinical features Multiple hyperpigmented macules on (a) oral mucosa and tongue, (b) plantar surfaces, and (c) longitudinal melanonychia.

A 76-year-old man with a medical history significant for chronic kidney disease, type 2 diabetes mellitus, hypertension, ischemic cardiopathy, and prior tobacco use (10 pack-years) was referred for evaluation of mucocutaneous hyperpigmentation present for five years. On physical examination, multiple brown to black pigmented macules, 3-5 mm in diameter, some confluent into larger patches, were observed on the distal phalanges of the hands, oral mucosa, tongue, glans penis, and plantar surfaces. The lesions were well-defined, flat, and had smooth surfaces (Figure 1). Dental amalgams were absent on physical examination and, therefore, excluded from differential diagnosis. 

**Figure 2:**
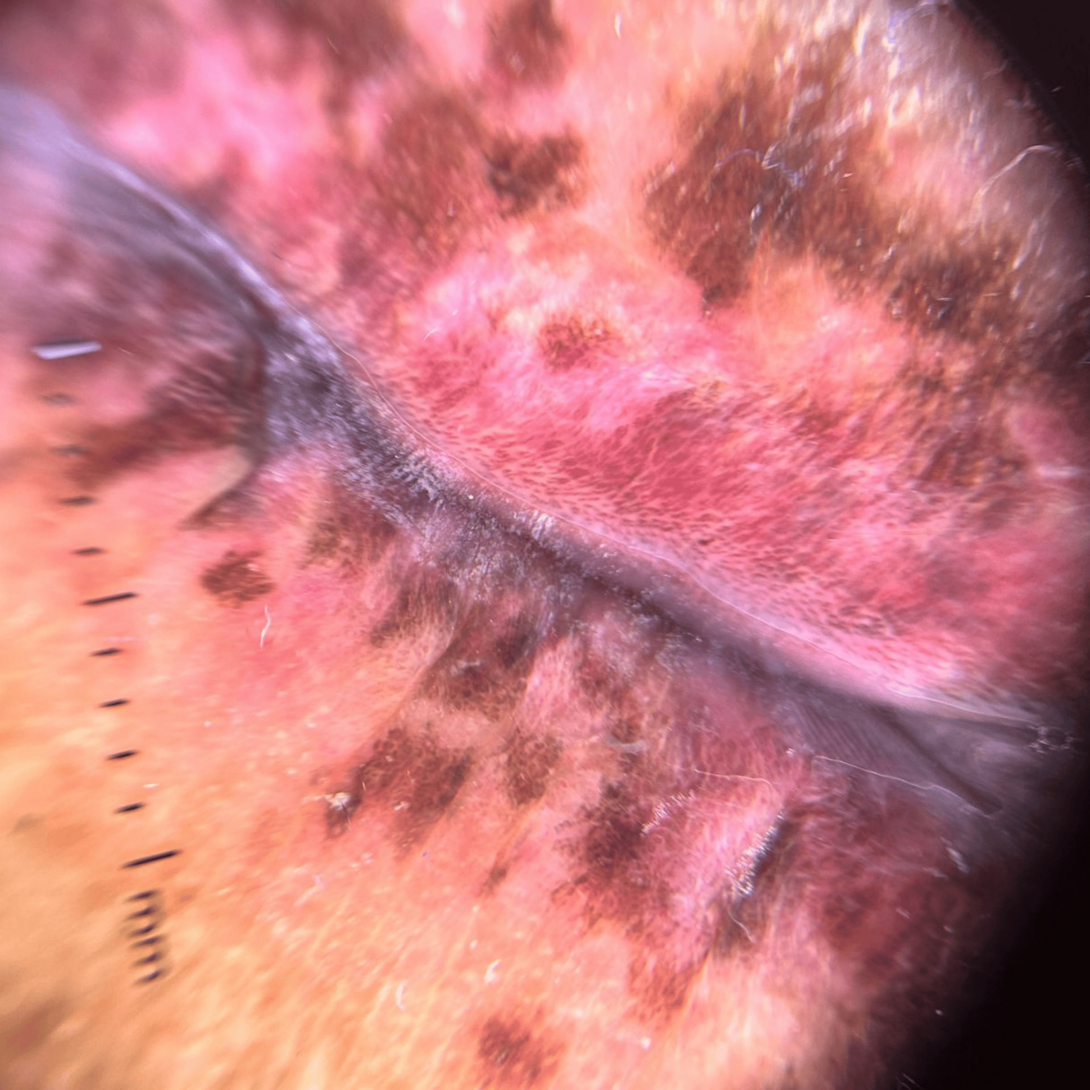
Dermoscopic findings Dermoscopy showing homogeneous brown pigmentation on the lips.

Dermoscopy revealed a diffuse dark brown homogeneous pigmentation on the lips (Figure 2).

**Figure 3:**
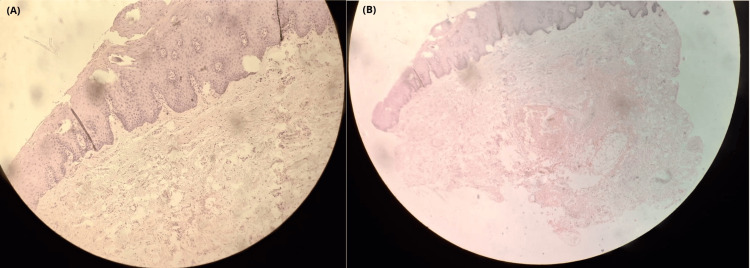
Histopathological findings Histopathology H&E-stained microphotographs (10×) demonstrated: (a) Slight epidermal thickening, increased pigmentation, and proliferation of melanocytes along the basal layer without nesting. (b) Numerous melanophages within the papillary dermis, and hyalinized collagen fibers consistent with solar elastosis.

A 5-mm punch biopsy of the labial mucosa demonstrated basal layer hyperpigmentation with melanophages in the superficial dermis, without melanocytic proliferation (Figure 3).

Endocrinological workup, including adrenocorticotropic hormone levels, excluded Addison’s disease and other endocrinological diseases (Table 1).

**Table 1:** Laboratory findings ACTH: adrenocorticotropic hormone; TSH: thyroid-stimulating hormone; CAE: carcinoembryonic antigen; AFP: alpha-fetoprotein

Test	Results	Reference value
TSH	5.76 mUI/L	0.40-5.10 mUI/L
T4T	8.21 ug/dl	4.5-12.5 ug/dl
T4F	1.24 ug/dl	0.89-1.76 ng/dl
T3T	108 ng/dl	84-172 ng/dl
T3F	3.04 pg/ml	2.4-5.6 pg/ml
Cortisol	15.3 ug/dl	4.6-24 ug/dl
ACTH	10.5 pmol/L	0-35 pmol/L
CAE	2.46 ng/ml	0-4.8 mg/ml
AFP	1.57 ng/ml	0-10 ng/ml

Gastrointestinal evaluation including endoscopy and colonoscopy ruled out polyps, excluding Peutz-Jeghers syndrome. Based on the clinical, dermoscopic, histopathological findings and absence of systemic involvement, the diagnosis of LHS was made.

## Discussion

LHS is an uncommon disorder, with approximately 200 cases reported in the literature [3,4]. It presents with acquired hyperpigmented macules involving the oral mucosa, lips, tongue, and sometimes genitalia, acral skin, and rarely the face [5,6]. Longitudinal melanonychia is often present and serves as a supportive diagnostic clue [3]. Despite its striking clinical features, LHS is benign and not associated with systemic disease or malignant transformation [7].

The clinical challenge lies in distinguishing LHS among other causes of acquired oral melanotic macules. Ferreira et al. conducted a systematic review of oral pigmented lesions in syndromic patients and found LHS to be the most frequent among syndromic causes of oral macules, followed by Peutz-Jeghers syndrome [1]. Peutz-Jeghers syndrome remains the most important differential diagnosis due to its association with gastrointestinal polyposis and increased risk of gastrointestinal and extraintestinal malignancies [1,3].

Other systemic conditions presenting with mucocutaneous pigmentation include Addison’s disease, LEOPARD syndrome, Carney complex, McCune-Albright syndrome, and Nelson’s syndrome [1,6,7-10]. Exogenous factors such as tobacco use, dental amalgams, medications (e.g., antimalarials, minocycline), and physiologic pigmentation in darker phototypes must also be excluded [8]. Histopathology in LHS typically demonstrates basal layer hyperpigmentation without melanocytic proliferation, helping differentiate it from melanocytic neoplasms such as mucosal melanoma [4,5,9,10].

Treatment of LHS is not mandatory, as the disorder is benign and cosmetic in nature [3]. Nonetheless, various modalities have been reported for esthetic improvement, including Q-switched Nd:YAG and Alexandrite lasers, as well as cryosurgery, with favorable results [3,8]. Patient education regarding the benign course of the disease and sun protection, particularly following laser therapy, is essential [3].

Accurate recognition of LHS is critical to avoid unnecessary invasive procedures, reduce patient anxiety, and differentiate it from potentially life-threatening conditions [3,7,10].

## Conclusions

LHS in a benign acquired disorder that represents one of the most common syndromic causes of acquired melanocytic macules. No malignancy has been associated with this entity, but more information should be obtained on this matter since etiology and pathogenesis remain poorly understood. Establishing an accurate diagnosis not only prevents unnecessary investigations but also reassures patients regarding its benign nature.
